# Observation of the influences of diosgenin on aging ovarian reserve and function in a mouse model

**DOI:** 10.1186/s40001-017-0285-6

**Published:** 2017-10-18

**Authors:** Mingjie Shen, Cong Qi, Yan-Ping Kuang, Yang Yang, Qi-Feng Lyu, Hui Long, Zhi-Guang Yan, Ying-Yu Lu

**Affiliations:** 1 Department of Gynecology and Obstetrics, Shu Guang Hospital Affiliated to Shanghai University of Traditional Chinese Medicine, Road Zhangheng No. 528, Pu Dong District, Shanghai, 201203 China; 2 Department of Assisted Reproduction, Shanghai Ninth People’s Hospital Affiliated to Shanghai Jiao Tong University of Medicine, Road Zhizaoju No. 639 Huangpu District, Shanghai, 200000 China; 3 Laboratory of Immunology and Virology, Experiment Center for Science and Technology, Shanghai University of Traditional Chinese Medicine, Shanghai, China

**Keywords:** Diosgenin, Ovarian reserve, Aging, Mouse model

## Abstract

**Background:**

The aim of this study was to investigate the impact of diosgenin, an important monomer of sapogenins in yams, on ovarian reserve in a natural aging mice model.

**Study design:**

This randomized controlled trial included 60 9-month-old C57 naturally aging female mice. Twenty-one mice were assigned to the dio group and were fed a single dose of diosgenin (200 mg/kg/day) suspended in 0.3% CMC. Twenty mice were assigned to the DHEA group and were fed a single dose of DHEA (1.25 mg/kg/day) suspended in 0.3% CMC. The remaining 20 mice were assigned to the old control group and were fed a single dose of 0.3% CMC. Three months later, the reproductive performance of these female mice was determined by evaluating ovarian follicles and oocyte number and quality in IVF and comparing age-matched and young controls. The impact of NOBOX, GDF9 and BMP15 mRNA expression was also evaluated.

**Results:**

Diosgenin improves ovarian reserve in naturally aging mice in terms of increasing the number of primary follicles (*P* < 0.05) and serum levels of AMH (*P* < 0.05).

**Conclusions:**

Diosgenin could counteract age-associated ovarian dysfunction by improving the ovarian reserve in a natural aging mice model.

## Background

Many women in the workforce postpone their childbearing, but their aging ovaries constitute a robust negative factor when they attempt to conceive [1]. Age-associated infertility has also been a great challenge to doctors using assisted-reproductive technologies. It has been reported that age is the strongest predictor of ovarian response and pregnancy rate [2]. Most evidences support the concept that women are born with a fixed number of oocytes that cannot regenerate and are depleted with age [3]. However, in contrast, few studies have suggested the presence of germ stem cells that could potentially replace lost follicles [4, 5]. It is known that the ovarian primordial follicle reserve is established during fetal development and that after birth the primordial follicle pool is continuously activated, while the rest of the pool remains quiescent for years or even decades until menopause [6]. With the remarkable decline in ovarian reserve, women by the age of 30 years retain only 12% of their ovarian reserve; and by the age of 40 years only 3% [7]. The extremely low pregnancy rate of women over the age of 40 years results from the decline in primordial follicle numbers. This parallels the decrease in healthy growing small antral follicles (an important dynamic reserve for ovulation) and the deterioration of oocyte quality [8].

Since the significant diminution in the ovarian reserve is a physiologic and anatomic fact that older women have to face, there is a need for greater attention on efficiency related to follicular development. Data indicate that the overwhelming majority of follicles undergo atresia at a relatively early stage of follicular development. Improving the efficiency of follicular development and preventing more primordial follicles from attaining the fate of atresia is the strategy we can use, in principle, to protect against ovarian aging and thereby prolong the reproductive life span. This is the current focus of our study.

The molecular control of oogenesis is complicated. A vast number of ovarian factors regulate this process including members of the transforming growth factor-beta (TGF-β) family, which control the growth and differentiation of somatic and germ cells. Growth differentiation factor-9 (GDF-9) and bone morphogenetic protein-15 (BMP-15) of the TGFβ family are well-known ovarian factors that regulate the process of follicular development and are secreted by oocytes [9]. With respect to the activation and suppression of primordial follicles, the PTEN/PI3k pathway plays a vital role [10, 11]. A recent study has indicated that newborn ovary homeobox-encoding gene (NOBOX), one of the oocyte-specific transcription factors, is an important player in the activation of primordial follicles and the transition to primary follicles. Without NOBOX, the majority of ovarian follicles are arrested at the primordial stage, and oocytes degenerate and do not develop beyond single-layered cuboidal primary follicles. NOBOX expression can also influence other important oocyte transcripts such as GDF-9, BMP-15 and Oct4 that occupy roles at different follicular stages [12].

Diosgenin ([25R]-5-spirosten-3β-ol) is a naturally occurring steroidal saponin that is present in a variety of plants including *Dioscorea* species, fenugreek and *Costus speciosus* [13]. Diosgenin has been initially acknowledged to be the starting material for the synthesis of a number of steroid hormones [14] and has now been reported to exert antiproliferative and proapoptotic actions on rheumatoid arthritis synoviocytes [15]. In addition, diosgenin exhibits other biological activities such as anticancer activity [16–18], antiviral activity [19] and antiinflammatory activity [20]. Diosgenin even shows potentially practical applications in the clinical treatment of heart disease [21]. However, diosgenin has never been reported to demonstrate actions with respect to improving ovarian function. Thus far, as an important monomer in yams (which has been acknowledged to improve women’s ovarian function in traditional Chinese medicine, and is included by millions of Chinese women in their diets), there is the distinct possibility that diosgenin might improve ovarian function.

In this study, we hypothesize that diosgenin counteracts age-associated ovarian dysfunction and improves the ovarian reserve in a mouse model of reproductive aging. We performed a 3-month administration of diosgenin in mice, to test the effects of diosgenin on improving overall reproductive function.

## Methods

### Animals and treatments

Female C57 mice were purchased at 9 months of age from the Vital River Laboratory Animal Technology Co., Ltd. The animals were housed under 12:12-h light–dark cycle conditions in a specific pathogen-free animal facility located at the Experimental Animal Center of Shanghai University of Traditional Chinese Medicine, China. The protocol of this study was approved by the Institutional Animal Committee. Mice were randomly divided into three groups: old control group, mice were fed 0.3% sodium carboxymethyl cellulose (CMC; purchased from Yuanye Bio-Technology Co., Ltd., Shanghai, China); DHEA group, mice were fed 1.25 mg/kg/day of dehydroepiandrosterone (DHEA; purchased from General Nutrition Center Inc., Pittsburgh, PA, USA), suspended in 0.3% CMC; dio group, mice were fed diosgenin (99% purity; purchased from Yuanye Bio-Technology Co., Ltd., Shanghai, China) at 200 mg/kg/day, suspended in 0.3% CMC. These treatments were intragastrically administered in each group once daily. Following treatment with diosgenin or DHEA for 3 months, some of the mice were randomly chosen to assess the ovarian reserve, ovarian response and oocyte quality with in vitro fertilization (IVF); while the remaining mice were used for breeding and evaluating litter size. Other mice were used to assess the expression of genes related to follicular development. Young mice at the age of 2–3 months served as controls (young control group) and were purchased from the Vital River Laboratory Animal Technology Co., Ltd.

### Ovarian serial sectioning and quantification of follicle counts

Ovaries were randomly collected from mice in the different groups (dio, DHEA, old control and young control groups). After immersion in 10% neutral-buffered formalin for at least 1 week, the tissues were dehydrated and embedded in paraffin wax and serially sectioned. Serial sections (5 µm) of each ovary were orderly aligned on glass microscope slides, stained with hematoxylin and eosin Y, and analyzed for the numbers of follicles at four different developmental stages using every fifth section with a random start in the first five sections. The total number of follicles per ovary was calculated by combining the counts in every fifth section throughout each entire ovary.

The follicles were categorized as primordial, primary, secondary, antral or pre-ovulatory, according to a previous study [22]. Follicles were classified as primordial if these contained an oocyte surrounded by a single layer of squamous granulosa cells, and were classified as primary if these were surrounded by a single layer of cuboidal granulosa cells. Secondary follicles were identified as having more than one layer of granulosa cells with no visible antrum. Antral follicles had small areas of follicular fluid (antrum), while pre-ovulation follicles had a single large antral space and cumulus oophorus.

### Anti-Mullerian hormone (AMH) measurements

A mouse AMH kit (US Biological Life Sciences, 23452, USA) was used to determine plasma AMH levels through enzyme-linked immunosorbent assay (ELISA). The analytical sensitivity of the kit was 0.05 ng/mL, and the standard curve spanned the range from 0.1 to 40 ng/mL.

### Oocyte retrieval

Female mice from the different groups were superovulated by injecting 10 IU of equine chorionic gonadotropin (eCG), followed by the administration of 10 IU of human chorionic gonadotropin (hCG) after 48 h. Female mice were humanely killed (CO_2_ overdose) 16 h after hCG injection and the oviducts were collected. The cumulus–oocyte complexes (COCs) were released from the ampullar region of each oviduct by puncturing the oviduct with a 28-gauge needle affixed to a 1-mL syringe, and these were collected by flushing the oviducts with human tubal fluid (HTF; EmbryoMax^®^, Millipore, USA). COCs were transferred and cultured in KSOM medium (Millipore, USA) with 5% CO_2_ at 37 °C and stored until use.

### IVF and embryo culture

To obtain sperm for IVF, 12-week-old male ICR mice were euthanized by cervical dislocation and epididymides were collected by dissection. Then, the epididymides were placed in the central well of an IVF dish with HTF medium. After making five to seven longitudinal cuts on each epididymis using a needle-affixed syringe, the epididymides were incubated for 20 min at 37 °C with 5% CO_2_ in compressed air to allow for sperm dispersion. The sperm suspensions were incubated for 1 h at 37 °C with 5% CO_2_ air to allow for capacitation. For IVF, MII oocytes were inseminated with 2 × 10^4^ sperms in a droplet of 150 µL of HTF medium for 4.5 h. The fertilized oocytes were subsequently cultured in a drop of 20 μL of KOSM medium at 37 °C with 5% CO_2_ in compressed air and high humidity. The development of the fertilized oocytes was monitored under an inverted microscope (Motic AE2000TRI) for the formation of two-cell-, four-cell-, morula-, and blastocyst-stage embryos at various intervals for up to 5 days.

### Gene expression by real-time PCR

Ovaries were lysed using TRIzol reagent (Invitrogen, Carlsbad, CA, USA) and DNA was reverse transcribed. The mRNA levels of three important genes related to follicle growth were measured by real-time PCR (RT-PCR) using iQSYBR green reagent (Qiagen, Valencia, CA, USA) and a Continuous Fluorescence Detection System (MJ Research Inc., Waltham, MA, USA). The mRNA expression was normalized to that of hypoxanthine-guanine phosphoribosyltransferase (HGPRT) for each sample, and the fold changes for each gene were calculated against those in normal mice. The details are shown in “Statistical analysis”.

## Statistical analysis

Experimental data were presented as mean ± standard deviation (SD). Data were obtained from three independent experiments, with three replicates per experiment. Data were evaluated by one-way analysis of variance (ANOVA) with the Tukey HSD post hoc test for comparisons between groups. *P* < 0.05 was considered to be statistically significant.

### Diosgenin increases primary follicle numbers

**Fig. 1:**
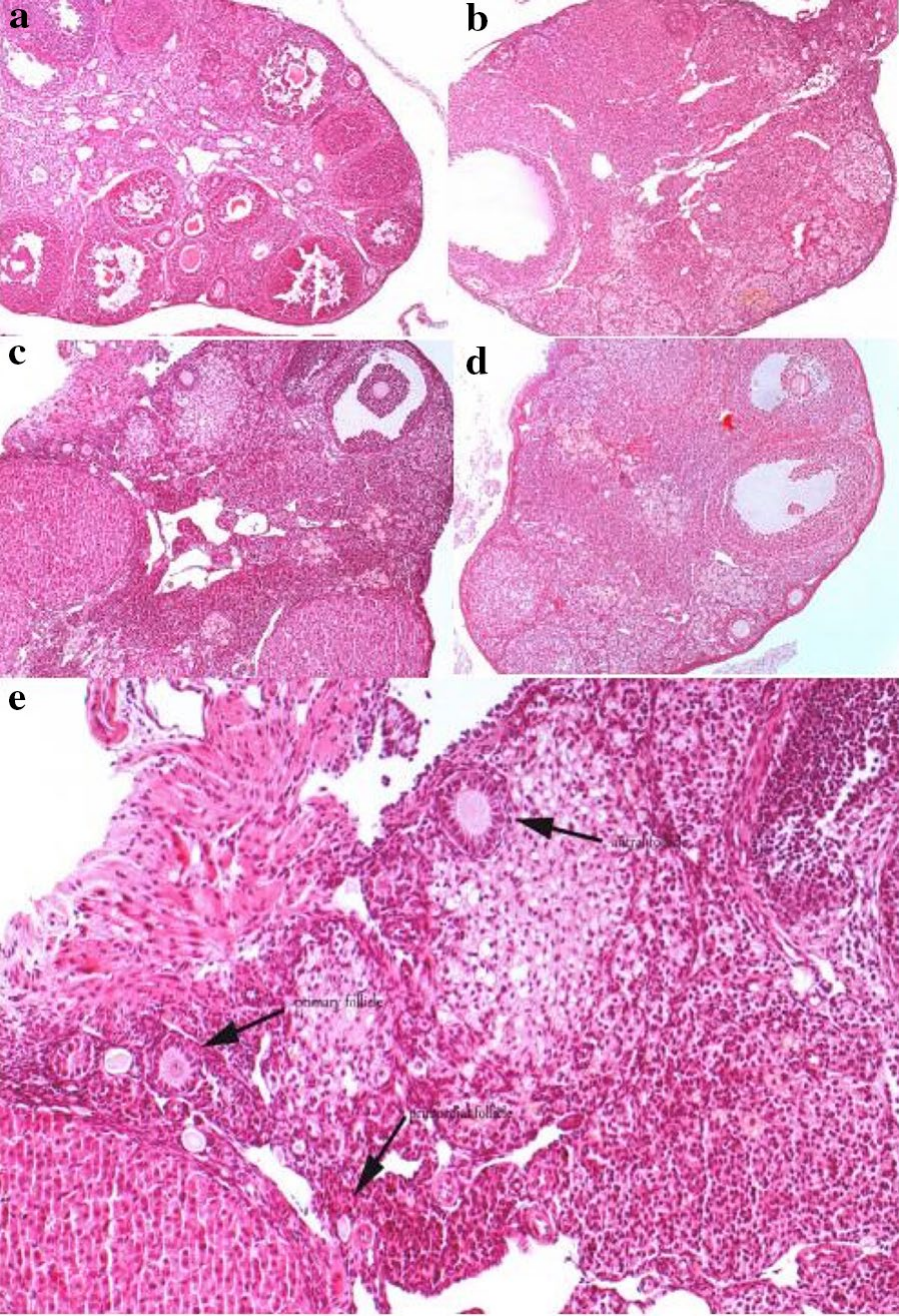
Typical photomicrographs (× 100) of the mice in the young control group (**a**), old control group (**b**), dio group (**c**) and DHEA group (**d**), and the details of the dio group (**e**)

**Fig. 2:**
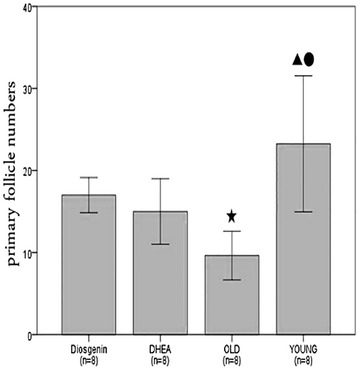
Primary follicle numbers. Bars with ^★^ superscripts indicate that the value is significantly different compared with the value for the dio group (^★^ *P* < 0.05); (2) bar with ^●^ superscript indicates that the value is significantly different compared with the value for the OLD group (^●^ *P* < 0.05); (3) bars with ^▲^ superscripts indicate that the value is significantly different compared with the value for the DHEA group (^▲^ *P* < 0.05). Values are mean ± SD

Young C57 females exhibited a large number of primary and primordial follicles, as well as secondary, antral and mature follicles. At the same time, the number of follicles was remarkably reduced in every category of aging female mice (old control group). Age-matched female mice treated with diosgenin exhibited more primary and primordial follicles, compared with aging controls. Although the number of primary follicles in the dio group was statistically greater than that in the old control group, the difference in the number of primordial follicles was not statistically significant (albeit a tendency for an increase was observed). However, diosgenin did not increase the number of growing or pre-ovulatory follicles after 3 months of treatment. Furthermore, the number of atretic follicles did not differ between the old control and the dio groups (Figs. 1 and 2, Table 1).

**Table 1:** Follicle counts of ovarian specimens from diosgenin, DHEA, old and young groups

Group	*n*	Primordial	Primary	Secondary	Antral	Pre-ovulation	Atretic
Diosgenin	8	18.63 ± 10.06	17.00 ± 2.14	6.63 ± 2.26	6.75 ± 2.92	2.13 ± 2.64	1.88 ± 2.23
DHEA	8	10.50 ± 3.38	15.00 ± 4.00	8.50 ± 3.89	7.25 ± 2.82	0.50 ± 0.53	1.13 ± 1.36
Old	8	8.88 ± 4.88	9.63 ± 2.97^★^	5.88 ± 2.64	6.25 ± 3.01	1.50 ± 1.93	3.63 ± 1.92^▲^
Young	8	27.38 ± 8.62^▲●^	23.25 ± 8.29^▲●^	30.25 ± 8.92^★▲●^	65.50 ± 17.39^★▲●^	10.63 ± 3.46^★▲●^	0.50 ± 0.76^●^
*F*		10.979	10.321	40.821	84.182	30.431	5.272
*P*		< 0.001	< 0.001	< 0.001	< 0.001	< 0.001	0.005

(1) Values with ^★^ superscripts are significantly different compared with the value for the dio group (^★^ *P* < 0.05); (2) values with ^●^ superscript are significantly different compared with values for the OLD group (^●^ *P* < 0.05); (3) values with ^▲^ superscript are significantly different compared with values for the DHEA group (^▲^ *P* < 0.05). Values are mean ± SD

### Diosgenin increased AMH serum levels

**Fig. 3:**
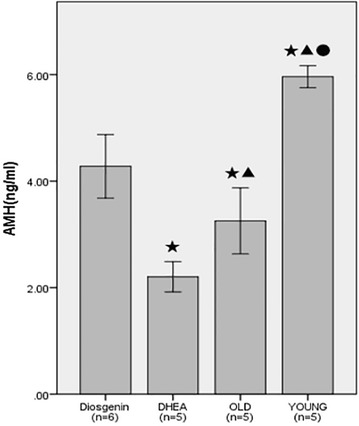
Serum AMH levels in mice. Bars with ^★^ superscripts indicate that the value is significantly different compared with the value for the dio group (^★^ *P* < 0.05); (2) bar with ^●^ superscript indicates that the value is significantly different compared with the value for the OLD group (^●^ *P* < 0.05); (3) bars with ^▲^ superscripts indicate that the value is significantly different compared with the value for the DHEA group (^▲^ *P* < 0.05). Values are mean ± SD

Anti-Mullerian hormone serum levels in aging mice (old control group) were significantly decreased compared with the young control group, while AMH levels were markedly higher in aging mice treated with diosgenin (dio group) compared with age-matched mice in the old control group and the mice treated with DHEA (Fig. 3).

### Diosgenin tends to increase the number of oocytes retrieved and improves the fertilization rate in IVF

The number of oocytes retrieved, MII oocytes and fertilized oocytes in aging mice treated with diosgenin tended to increase, compared with the old control and DHEA groups, but the difference was not statistically significant (Table 2).

**Table 2:** Effects of oocyte retrieval and fertilization in vitro (IVF)

Group	*n*	No. of oocytes per mouse	No. of MII oocytes per mouse	No. of fertilized oocytes per mouse	Fertilization rate
Dio	5	2.80 ± 2.59	1.80 ± 1.64	1.60 ± 1.52	0.73 ± 0.43
DHEA	5	1.00 ± 1.22	0.80 ± 0.84	0.40 ± 0.55	0.30 ± 0.45
Old	5	0.80 ± 1.10	0.60 ± 0.89	0.40 ± 0.55	0.30 ± 0.45
Young	5	12.00 ± 2.74^★▲●^	9.80 ± 2.86^★▲●^	9.60 ± 2.61^★▲●^	0.98 ± 0.03
*F*		33.369	31.199	40.577	3.887
*P*		< 0.001	< 0.001	< 0.001	0.029

(1) Values with ^★^ superscripts are significantly different compared with the value for the dio group (^★^ *P* < 0.05); (2) values with ^●^ superscript are significantly different compared with the value for the Old group (^●^ *P* < 0.05); (3) values with ^▲^ superscript are significantly different compared with the value for the DHEA group (^▲^ *P* < 0.05). Values are mean ± SD

### Diosgenin influences the gene expression of NOBOX and GDF-9

**Fig. 4:**
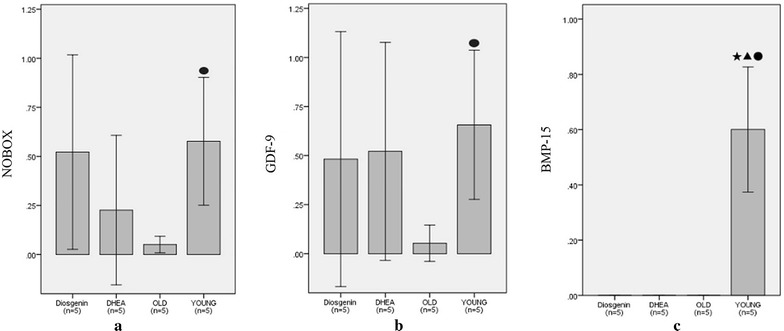
Three oocyte-specific gene expression in ovaries. **a** The relative mRNA levels of NOBOX, **b** the relative mRNA levels of GDF-9, and **c** the relative mRNA levels of BMP-15. (1) Bars with ^★^ superscripts indicate that the value is significantly different compared with the value for the dio group (^★^ *P* < 0.05); (2) bars with ^●^ superscript indicates that the value is significantly different compared with the value for the OLD group (^●^ *P* < 0.05); (3) bars with ^▲^ superscripts indicate that the value is significantly different compared with the value for the DHEA group (^▲^ *P* < 0.05). Values are mean ± SD

The expression of NOBOX, GDF-9 and BMP-15 decreased in aging mouse (old control group) ovaries compared with the young mice (*P* < 0.05). However, in ovaries from aging mice treated with diosgenin, NOBOX and GDF-9 gene expression revealed a tendency to increase; but the increase was not significant. This was also the case for aging mice treated with DHEA with regard to the expression of GDF-9. Neither diosgenin nor DHEA influenced the expression of BMP-15 (Fig. 4).

## Discussion

Our results indicate that diosgenin can increase the number of primary follicles, and a tendency for the number of primordial follicles to increase was observed, thereby improving the ovarian reserve in aging mice. Diosgenin revealed a tendency to increase oocyte retrieval and fertilization rate in vitro. This suggests that diosgenin can slow ovarian aging and improve the response to gonadotropin by aging ovaries in IVF by replenishing the reduction in the follicular pool.

According to classical gynecologic theory, female mammals are born with a fixed number of oocytes that continuously reduces until these animals are no longer able to conceive [23]. The potential presence of ovarian stem cells remains controversial, and most scholars unequivocally state that oocytes in female mammals cannot be renewed.

In today’s society, more women continue to delay their childbirth; therefore, when a woman first wishes to conceive at the age of 30 or even 40 years, the reduction in oocyte quality and quantity can cause problematic issues. In terms of classic gynecologic theory, the tendency for the decline in fertility is inevitable. However, this does not mean that we have no ability to delay ovarian aging. Our results revealed that diosgenin treatment contributed to the improvement in the ovarian reserve of naturally aging mice by increasing the number of primary follicles, when compared with aging controls.

Anti-Mullerian hormone is produced by granulosa cells of developing pre-antral and small antral follicles [24]. Serum AMH levels are widely used to estimate the size of the ovarian reserve and are regarded as a sensitive indicator of ovarian function in the clinic [25]. In our study, serum AMH levels, together with the quantification of follicle numbers, indicated that the ovarian reserve in aged mice could be improved to some extent. In addition, IVF results revealed that diosgenin tended to increase the number of oocytes retrieved, MII oocytes and fertilized oocytes in aging mice, although our interpretations were limited by the small sample size. The increased number of MII oocytes and fertilized oocytes in the dio group, compared to the old control group, indicates that diosgenin could improve both the number and quality of oocytes. Therefore, diosgenin appeared to protect against ovarian aging and contributed to the improvement in IVF results in aging mice.

Primordial follicles constitute the total reservoir of germ cells available during the entire female reproductive life span. Despite this, the mechanism(s) for the formation and activation of primordial follicles are not fully understood, although GDF9, BMP15 and NOBOX are known to be involved in these processes.

GDF9 and BMP15 are important members of the TGF-β family and are produced exclusively by growing oocytes in the ovary, playing a vital role in regulating the development of oocytes and proliferation and differentiation of ovarian granulosa cells. In GDF9-null mice, folliculogenesis does not progress beyond the primary stage [26], while BMP15-null mice have impaired oocyte maturation that leads to infertility [27]. In our study, GDF9 and BMP15 were significantly different between the young and old control groups, which indicate that the reduced ovarian reserve observed with decreasing pre-antral follicle count was associated with the reduction in the expression of GDF9 and BMP15 in aging ovaries. NOBOX is an oocyte-specific homeobox gene expressed in germ cell cysts, as well as in primordial and growing oocytes. The proper expression of NOBOX is crucial for ovarian development in both mice and humans [28–36]. Our data also confirmed these concepts. The reduced expression of NOBOX in the ovaries was commensurate with ovarian aging and the reduction in the ovarian reserve.

Our results revealed that there were increasing numbers of primary follicles and a growth trend of primordial follicles in the dio group, compared with the old control group. Furthermore, this suggests that diosgenin appears to stimulate the formation and activation of primordial follicles. Consequently, we hypothesized that the ovarian reserve could be improved in old mice treated by diosgenin. NOBOX was expressed in oocytes, and GDF9 was expressed in granulosa cells in growing follicles. Due to increasing numbers of primary follicles and the growth trend of primordial follicles in the dio group, the elevated level of NOBOX and GDF9 was understandable. Interestingly, the expression of GDF9 was also increased in mice in the DHEA group. Despite this, there was a slight non-significant increase in the number of primordial, primary and secondary follicles in the DHEA group. DHEA appeared to increase the number of pre-antral follicles to some extent, but did not enhance the retrieval of MII oocytes or promote the quality of oocytes in our study. This might be related to the reduced expression of NOBOX and increased expression of GDF9 in the DHEA group.

## Conclusions

The present study supports our hypothesis that diosgenin increases the number of primary follicles, leading to the promotion of ovarian reserve in a naturally aging mouse model. Although our research shows that diosgenin benefits ovarian reserve in aging mice, data in this mouse model may not be directly extrapolatable to human ovarian reserve. Hence, more extensive research is needed if any clinic trials are to be attempted.
